# Editorial: Using Stress-Based Animal Models to Understand the Mechanisms Underlying Psychiatric and Somatic Disorders

**DOI:** 10.3389/fpsyt.2016.00192

**Published:** 2016-12-01

**Authors:** Stefan O. Reber, David A. Slattery

**Affiliations:** ^1^Laboratory for Molecular Psychosomatics, Clinic for Psychosomatic Medicine and Psychotherapy, University Ulm, Ulm, Germany; ^2^Department of Psychiatry, Psychosomatic Medicine and Psychotherapy, University Hospital Frankfurt, Frankfurt am Main, Germany

**Keywords:** stress, microbiome, animal models, glucocorticoids, prefrontal cortex, behavior, irritable bowel syndrome

**The Editorial on the Research Topic**

**Using Stress-Based Animal Models to Understand the Mechanisms Underlying Psychiatric and Somatic Disorders**

Chronic or repeated stress, particularly psychosocial stress, is an acknowledged risk factor for numerous affective and somatic disorders in modern societies. Thus, there is substantial evidence showing that chronic stress can increase the likelihood of major depressive disorder and anxiety disorders, as well as cardiovascular diseases, irritable bowel syndrome and pain syndromes, to name but a few, in vulnerable individuals. Although a number of pharmacological agents are available to treat such stress-related disorders, many patients do not respond to them, and those who do often report a number of side effects.

Consequently, a major emphasis in modern basic research is to uncover the underlying etiology of these disorders and to develop novel efficacious treatment strategies. Animal models can be utilized to unravel the biological mechanisms underlying specific disorders and test the efficacy of novel drugs. While animal models cannot mimic psychiatric and somatic disorders in their entirety, assessing the impact of certain risk factors for the disorders will facilitate the understanding of their etiology and treatment options. Consistent with this knowledge, and with clinical evidence purporting chronic stress to be a risk factor for such diseases, recent attempts have focused on the development of adequate stress paradigms. It is believed that psychosocial stress paradigms are more relevant to the human situation than non-social stress paradigms, because the vast majority of stressors reported by patients suffering from psychiatric or somatic disorders are social in nature. Therefore, there is a growing consensus that social stress paradigms are better placed to reveal the behavioral, neuroendocrine, or immunological mechanisms underlying chronic stress-induced pathology. However, in order to maximize our understanding of the mechanisms underlying stress-related disorders, various different models are necessary, including models employing physical (i.e., footshock) or pharmacological (e.g., corticosterone) stressors. Moreover, there is accumulating evidence suggesting that stress-induced gut dysbiosis and systemic low-grade inflammation play a causal role in the development of stress-associated somatic and affective disorders. Therefore, chronic/repeated stress models that result in reduced gut microbial diversity and/or promote chronic immune activation are of special relevance and importance.

In this research topic, we have collated novel research articles (four) and comprehensive review articles (five) from various leaders in the field of stress-based research. The overarching goal of the topic is to reveal how chronic/repeated stress models can help us to better understand the etiology of psychiatric and somatic disorders.

The first article describes a novel chronic unpredictable stress (CUS) protocol, which was developed for C57BL/6 mice. Given the unreliability of commonly utilized 4-week-long CUS protocols in C57BL/6, Monteiro et al. developed an 8-week-long CUS protocol that results in classical stress-related outcomes, such as increased adrenal weight and increased anxiety- and depressive-like behavior. Their results suggest that this 8-week protocol can be used to assess the underlying mechanisms of stress-related alterations in C57BL/6 mice.

Chronic stressor exposure can also be mimicked by treating rodents chronically with corticosterone. Here, Kinlein et al. demonstrate that non-invasive corticosterone administration *via* the drinking water for 28 days (25 μg/ml) results in a dysregulated response to a subsequent acute stressor. While corticosterone-treated mice did not show a plasma corticosterone response to swim exposure, their central *c-fos* response to this acute stressor was actually increased in numerous brain regions. Taken together, these findings suggest that this model can be used to determine how altered HPA axis function contributes to central dysregulation to an acute stressor.

Another system disrupted by chronic/repeated stressor exposure is the sleep/wake cycle. In their study, Greenwood et al. assess the effect of repeated (22 days) contextual fear conditioning on behavior and the sleep/wake cycle to a subsequent acute foot shock. Their findings demonstrate that the increased anxiety-related behavior and disrupted sleep/wake behavior evoked by acute stress is enhanced by prior fear conditioning. Taken together, this study suggests that repeated stress increases the vulnerability to novel subsequent stressors.

The extent to which chronic stress interacts with HIV-1 viral proteins to impact behavior and neuroinflammatory processes is largely unknown, but of growing research interest. Addressing this question, Rowson et al. expose male and female HIV-1 transgenic rats to chronic stress in adolescence (or non-stressed controls) and reveal that the presence of the transgene affects behavior and microglial structure, particularly in females. However, the addition of stressor exposure did not further affect these alterations, suggesting that there is little interaction between stress and HIV-1 viral proteins.

In the second section of the research topic, complementing the original research articles, are five review articles covering a range of topics related to stress-based research. The first review article describes the use of basic rat models to gain a better understanding of the effect of chronic stress on myocardial sensitivity to ischemic injury. Eisenmann et al. suggest that acute stress decreases the sensitivity to myocardial ischemia–reperfusion injury, while chronic stress even increases myocardial sensitivity to such an injury.

Stress has been well documented to affect synaptic plasticity and cause dramatic alteration of the glutamatergic system. In their review article, Musazzi et al. summarize our current understanding of the biphasic adaptations of the glutamatergic system in the prefrontal cortex during stressor exposure. The implications of these adaptations for our understanding of the etiology, and possible treatment, of stress-related disorders are then discussed.

There has been a growing reliance on chronic psychosocial stress models in basic research, due to the belief that such models more accurately reflect the clinical situation. Langgartner et al. discuss the knowledge that such models have provided in relation to psychiatric and somatic disorders. They focus particularly on one such model, the chronic subordinate colony housing (CSC) paradigm, which causes numerous stress consequences, including increased anxiety-related behavior, spontaneous colitis, and HPA axis changes.

Finally, given the growing interest in the role of the brain–gut axis on affective behavior and somatic disorders, there are two reviews focusing on this topic. Gur et al. discuss the evidence revealing that although the gut microbiota is relatively resistant to change, at particular time points, such as early life, stress can affect the microbiota and lead to detrimental outcomes for parturition and infant neurodevelopment. Moloney et al. review current animal models of stress-induced visceral pain, which aim to reflect irritable bowel syndrome in patients. Their wide-ranging review describes the influence of a number of factors, such as stress, gender, gut microbiota and epigenetic changes, in the etiology and potential treatment of visceral pain.

Taken together, the articles collated in this research topic provide a broad overview of the role that stress-based animal models have in deepening our understanding of stress-induced disorders. Although not comprehensive, the articles cover models relevant for anxiety disorder, major depressive disorder, alcohol abuse, cardiovascular diseases, ulcerative colitis, and irritable bowel syndrome. Importantly, given the large comorbidity between such disorders, placing these articles together in one research topic ensures an overview about how multiple systems interact during chronic stressor exposure. Such multimodal research is becoming increasingly necessary to enhance our understanding of psychiatric and somatic disorders.

## Author Contributions

Both the authors contributed to the writing of the editorial and agreed with the final version.

## Conflict of Interest Statement

The authors declare that the research was conducted in the absence of any commercial or financial relationships that could be construed as a potential conflict of interest.

